# Tilted Endosseous Dental Implants in Atrophic Maxilla: A Clinical Prospective Study

**DOI:** 10.7759/cureus.112693

**Published:** 2026-07-15

**Authors:** Seymantika Sarvapriye, Srishti Rathore, Mohammad Sadique Ali, Nida Waris, Malik Hina, Aojashwi Jnawali, Rupinder Kaur, Sagel Rana, Gurkanwal Kaur Aurora, Nabiha N Syeda

**Affiliations:** 1 Oral and Maxillofacial Surgery, Buddha Institute of Dental Sciences & Hospital, Patna, IND; 2 Prosthodontics, Saraswati Dental College and Hospital, Lucknow, IND; 3 Prosthodontics, Career Institute of Dental Sciences and Hospital, Lucknow, IND; 4 Orthodontics and Dentofacial Orthopedics, B.P. Koirala Institute of Health Sciences, Dharan, NPL; 5 Dentistry, Baba Farid University of Health Sciences, Faridkot, IND; 6 Dentistry, Vokkaligara Sangha Dental College & Hospital, Bengaluru, IND; 7 Dentistry, Sri Balaji Dental College, Modinagar, IND

**Keywords:** atrophic maxilla, crestal bone loss, dental implants, endosseous implants, immediate loading, maxillary rehabilitation, osseointegration, sinus grafting alternative, tilted implants

## Abstract

Background

Atrophic edentulous maxillary arches present a significant surgical challenge for conventional implant placement owing to inadequate bone height, pneumatization of the maxillary sinus, and compromised bone quality in the posterior region. Traditional solutions, including sinus floor elevation, ridge augmentation, and bone grafting, carry inherent morbidity and procedural complexity.

Objective

This study aimed to evaluate the clinical and radiographic outcomes of tilted endosseous dental implants placed in atrophic maxillae, with specific assessment of osseointegration quality, crestal bone loss, periodontal parameters, and pain scores at three and six months post-operatively.

Methods

Thirty tilted endosseous implants were placed in atrophic maxillae of adult patients. Pre-surgical evaluation included cone-beam computed tomography (CBCT) and orthopantomography (OPG). Modified plaque index, gingival index, probing depth, crestal bone loss, osseointegration quality (Hounsfield units), and pain (McGill Pain Questionnaire and visual analog scale (VAS)) were assessed immediately post-operatively and at three and six months.

Results

Mean crestal bone loss at three and six months was 1.46 ± 0.53 mm and 1.75 ± 0.37 mm, respectively, for anterior implants and 1.25 ± 0.31 mm and 1.65 ± 0.35 mm, respectively, for posterior implants. A statistically significant difference (p < 0.05) was observed between anterior and posterior implants for crestal bone loss at both time points.

Conclusion

Tilted endosseous implants achieved a six-month survival rate of 93.3% (28 of 30 implants) in this cohort and represent a promising treatment option for the atrophic maxilla without requiring sinus augmentation. Larger, long-term prospective studies with a control group are warranted before broader claims of predictability can be made.

## Introduction

The edentulous state has an undesirable effect on oral health-related quality of life owing to impaired mastication, denture trauma, aesthetic concerns, and negative self-perception. Patients who have worn complete dentures for prolonged periods are often dissatisfied with prosthetic instability during speaking and eating. Dental implants have remarkably transformed the rehabilitation process and are considered a highly predictable treatment option for both partial and complete edentulism. However, in cases of atrophied edentulous maxillary arches, the placement of endosseous implants (>10 mm), particularly in the posterior region, is challenging owing to anatomic constraints, such as bone resorption, compromised bone quality, maxillary sinus pneumatization, and limited vertical space [1]. In the posterior maxilla, a minimum ridge height of 6-7 mm should be available for safe placement of implants shorter than 8 mm [2].

Several surgical methods have been developed to overcome these challenges, including sinus floor elevation, ridge augmentation, bone grafting, and Le Fort I osteotomy with interpositional grafting. However, these techniques involve considerable expenses, procedural complexity, prolonged treatment duration, and demands on patient compliance [3]. Sinus floor elevation has limited acceptance owing to risks of site morbidity, graft selection challenges, and post-operative discomfort [4]. Autologous bone harvested from sites such as the chin and calvaria has been widely used; however, these procedures are associated with complications, including graft loss and osteomyelitis [5]. To avoid these challenges, surgeons have proposed placing long tilted implants (>13 mm) in anatomic locations, such as the pterygoid region, zygomatic bone, or residual alveolar bone, to achieve high primary stability, improve anchorage, and optimize the anterior-posterior spread. This intervention thereby provides adequate molar support and reduces the cantilever extensions seen with axially placed implants [6].

Placing implants in a tilted orientation allows the use of longer implants with increased bone contact, eliminating the need for ridge augmentation and its associated post-operative complications [7]. Several studies have confirmed that tilting implants mesially, or placing them in direct contact with the anterior wall of the maxillary sinus without disrupting the sinus membrane, has demonstrated favorable outcomes [7]. In 1985, bone grafting was a standard practice for severely atrophied ridges; however, an alternative approach was later described by Fortin et al. [8], involving the placement of four implants along the anterior wall of the sinus in a tilted orientation to gain posterior biomechanical advantage. In patients with moderate-to-advanced maxillary ridge resorption, the residual bone forms a three-dimensional pyramid comprising the palate, anterior sinus cortical wall, and buccal plate. The authors have consistently placed tilted maxillary implants as an alternative to sinus grafting since 1992 to provide posterior support when resorptive patterns preclude axial alignment [8]. Multiple authors recommend placing tilted implants in combination with at least one additional implant in partially edentulous areas [4]. Tilting implants does not negatively affect load distribution during rehabilitation [1].

The tilted implant design necessitates the use of angled abutments, which increases stress on supporting implants and adjacent bone. Despite a 3.0- and 4.4-fold stress increase with 15° and 25° angled abutments, respectively, the resulting stress on bone remains within physiological limits when compared with straight abutments [9]. The rationale for tilting implants is to avoid sinus augmentation, bone grafting, and ridge augmentation while achieving high insertion torque for immediate loading [3]. This approach improves posterior prosthetic support; however, angulation should not exceed 45°, as angular forces may contribute to peri-implant bone resorption at the implant margin [9].

## Materials and methods

This prospective study enrolled adult patients presenting to the outpatient department (OPD) for replacement of missing maxillary teeth (anterior and posterior) with endosseous dental implants. The primary endpoint of this study was implant survival and success at six months. Secondary endpoints included crestal bone loss, periodontal parameters (plaque index, gingival index, probing depth), pain scores, and osseointegration quality (Hounsfield units) at three and six months. A total of 30 tilted endosseous dental implants (Bioline Dental Ltd., Migdal Tefen, Israel) were placed in atrophic maxillae. Before surgery, a detailed patient history was recorded; patients were informed of the potential risks and benefits of the study, and written informed consent was obtained in each patient's own language.

Inclusion criteria

Inclusion criteria were patients aged more than 18 years, compliance with the physical status classification I or II of the American Society of Anesthesiologists (ASA) [10], sufficient inter-occlusal space for implant placement, and atrophic maxilla (anterior and/or posterior) with severely resorbed ridges.

Exclusion criteria

Patients were excluded if they were classified as ASA III or IV [10], were unable to provide informed consent, had a history of sinus or maxillary grafting, or were on bisphosphonate medication.

Surgical protocol

**Figure 1:**
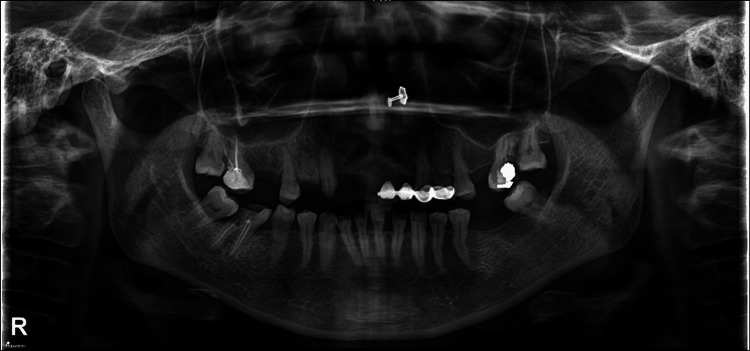
Pre-operative orthopantomograph (Case 1)

**Figure 2:**
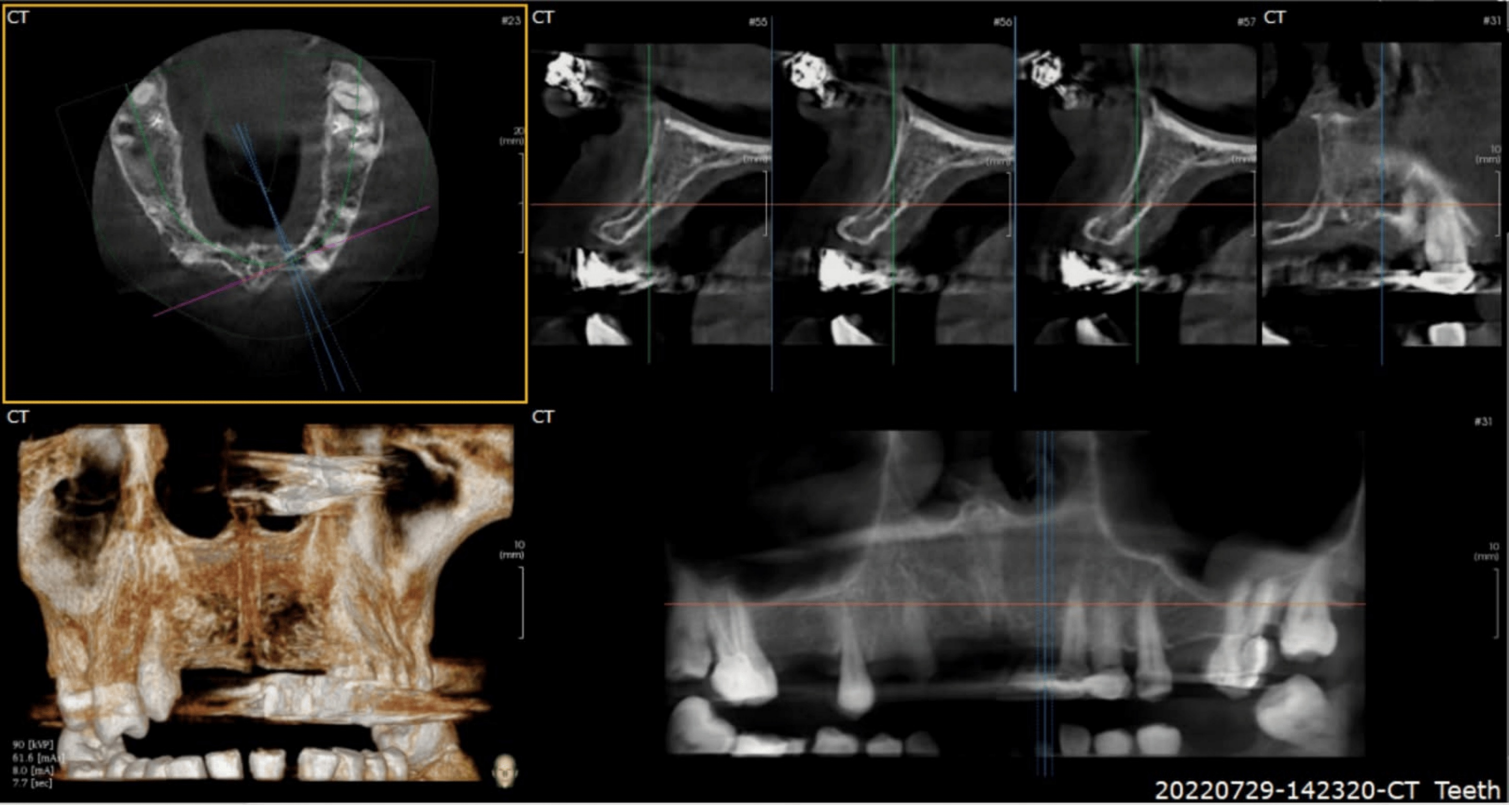
Pre-operative cone-beam computed tomography (Case 1)

**Figure 3:**
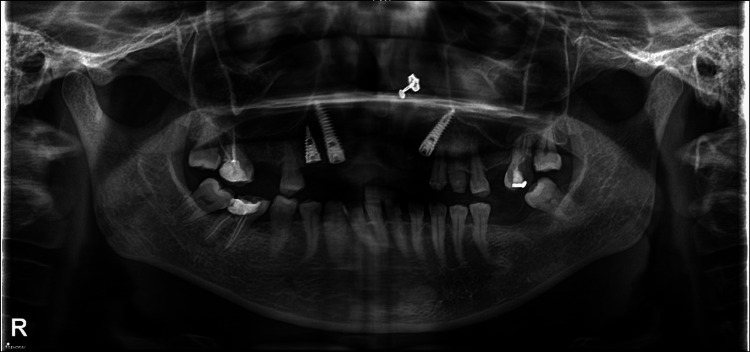
Post-operative orthopantomograph (Case 1)

A meticulous presurgical evaluation was performed for all patients. Pre-operative cone-beam computed tomography (CBCT) and orthopantomography (OPG) were obtained for Case 1, as shown in Figures 1-2. Full-thickness mucoperiosteal flaps were raised under local anesthesia via a mid-crestal incision, following the technique described by Krekmanov [11]. The entry point of the distal implant was determined by measuring the distance from the most anterior tooth using a dental caliper, guided by a CBCT scan or panoramic radiograph. The osteotomy angle and direction were planned after locating the anterior wall of the sinus using a pilot drill of 2.0 mm diameter. At this stage, a periapical radiograph was obtained to verify drilling accuracy and prevent perforating the sinus wall. Successive drilling was then performed according to the planned implant dimensions, followed by insertion of the distal tilted implant. The anterior implant was placed in the available bone between the most distal tooth and the posterior implant, positioned parallel to the adjacent tooth and approximately perpendicular to the bone crest, as determined by the surgeon. All implants were placed manually in a supra-crestal position. A post-operative OPG was obtained for Case 1, as shown in Figure 3.

Latency Phase (Case 1)

**Figure 4:**
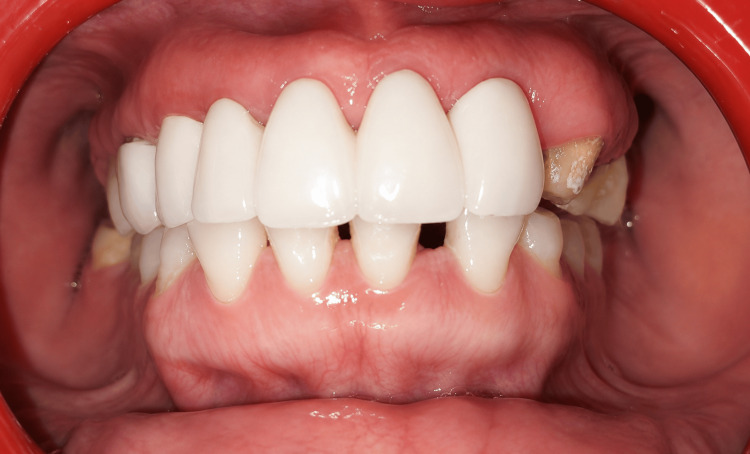
Post-rehabilitation intraoral view (Case 1)

Following a three-month healing period, prosthetic components were attached to the multiunit abutments. Impressions and bite registrations were obtained, and the definitive prosthesis was delivered (Figure 4).

**Figure 5:**
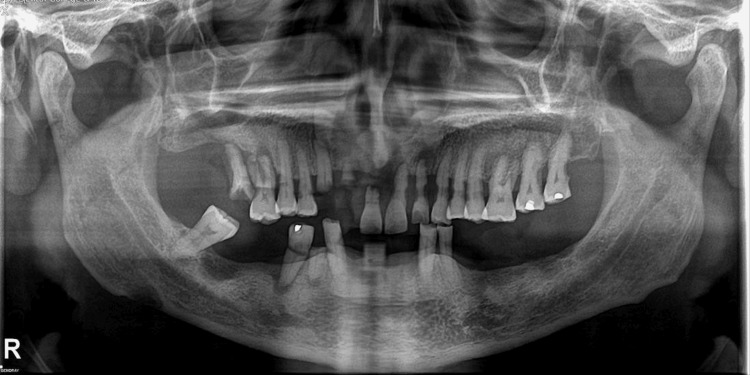
Pre-operative orthopantomograph (Case 2)

**Figure 6:**
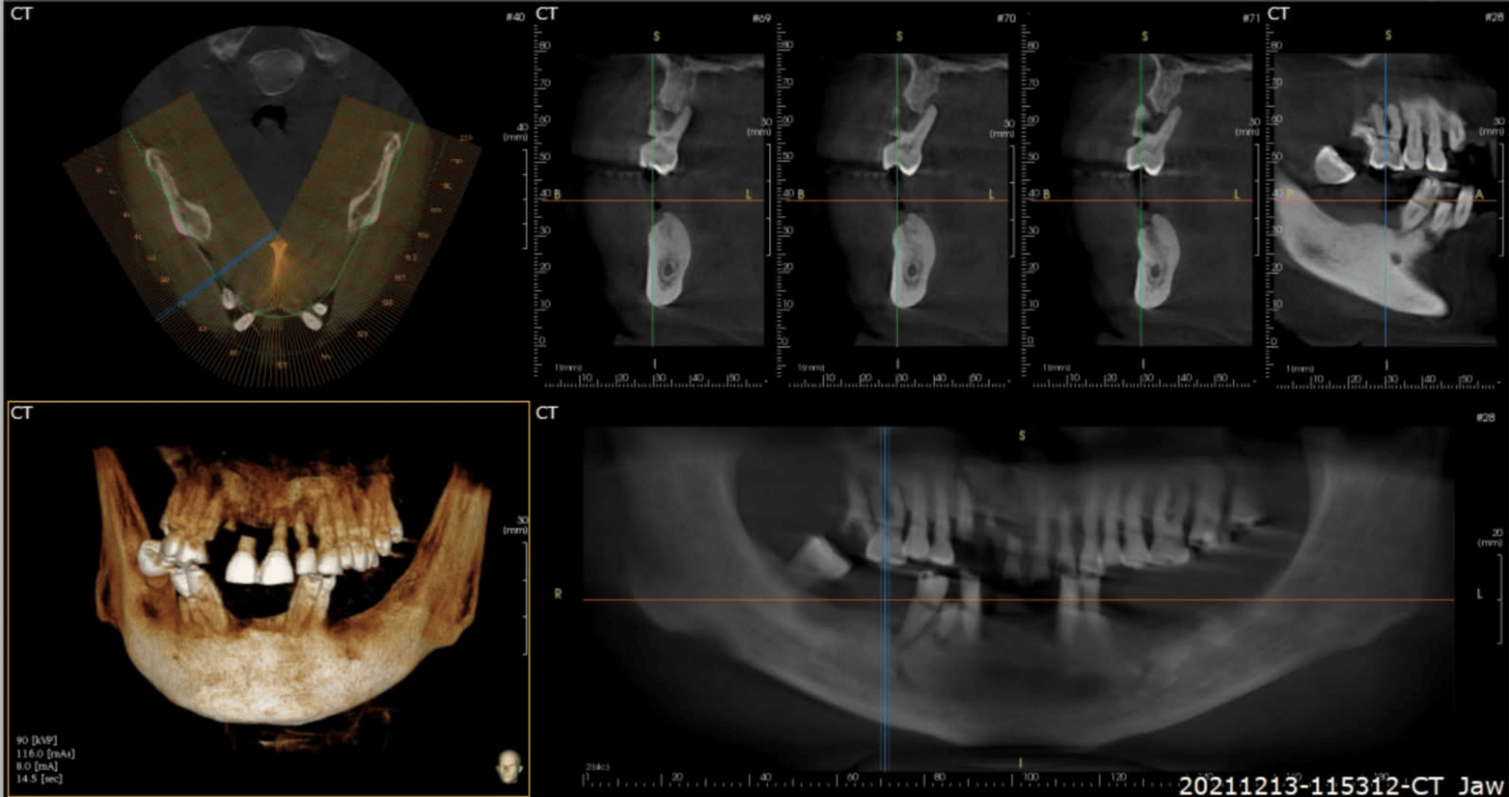
Pre-operative cone-beam computed tomography (Case 2)

**Figure 7:**
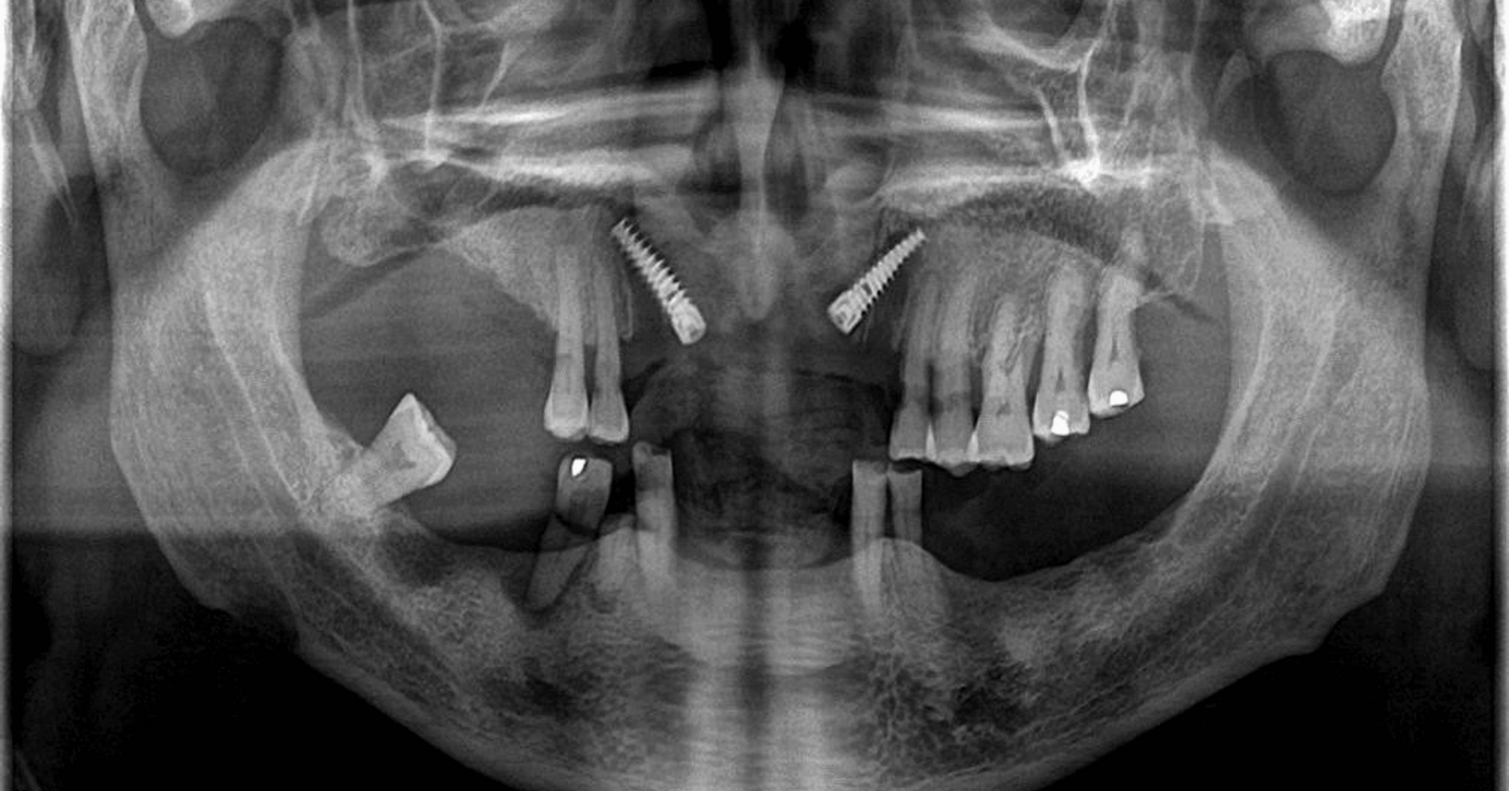
Post-operative orthopantomograph (Case 2)

The same protocol was followed for Case 2. Pre-operative OPG and CBCT were obtained, as shown in Figures 5-6. A post-operative OPG was obtained, as shown in Figure 7. In all cases, multiunit abutments were attached to the implants to avoid a second-stage surgery.

Latency Phase (Case 2)

**Figure 8:**
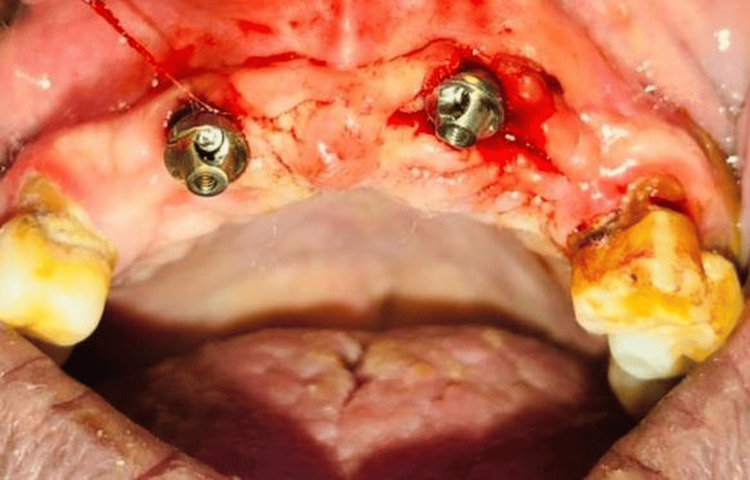
Intraoral clinical view following implant placement with multiunit abutments (Case 2)

**Figure 9:**
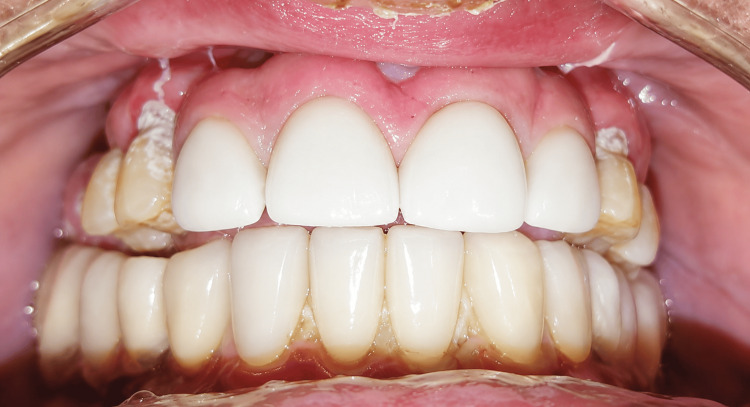
Post-rehabilitation intraoral view (Case 2)

Following a three-month healing period, prosthetic components were attached to the multiunit abutments, and the definitive prosthesis was delivered (Figures 8-9).

Methods of evaluation

Radiographic assessment using OPG and CBCT was performed to evaluate implant success and survival, bone density using Hounsfield units, and marginal bone loss. Periodontal examination included probing depth measured using the Community Periodontal Index of Treatment Need (CPITN) probe, modified plaque index [12], gingival index [13], and pain assessment using the McGill Pain Questionnaire [14] and visual analog scale (VAS) [15]. All parameters were assessed immediately post-operatively, at three months, and at six months.

Statistical analysis

Statistical analysis was performed using Statistical Product and Service Solutions (SPSS, version 23.0; IBM SPSS Statistics for Windows, Armonk, NY). Descriptive statistics, expressed as mean ± standard deviation, were calculated for all continuous variables. The chi-square test was used to evaluate associations between categorical variables, and a paired t-test was used to assess intergroup comparisons across time intervals. Statistical significance was set at p < 0.05. Implants placed within the same patient were not modeled as clustered observations in this analysis; this is addressed further in the Limitations section.

## Results

A total of 30 tilted endosseous dental implants (Bioline Dental Ltd., Migdal Tefen, Israel) were placed across the anterior and posterior maxillary regions in the study cohort. Two implants, one anterior and one posterior, placed in two different patients, became clinically mobile following prosthetic loading and were classified as failures. The overall six-month implant survival rate was 93.3% (28 of 30 implants).

The mean plaque index values at three and six months were 0.426 ± 0.64 and 0.71 ± 0.46, respectively, for anterior implants and 0.750 ± 0.44 and 0.37 ± 0.50, respectively, for posterior implants. The mean gingival index values at three and six months were 0.64 ± 0.49 and 0.78 ± 0.69, respectively, for anterior implants and 0.37 ± 0.49 and 0.68 ± 0.47, respectively, for posterior implants. The mean probing depth values at three and six months were 1.53 ± 0.45 mm and 1.85 ± 0.41 mm, respectively, for anterior implants and 1.65 ± 0.56 mm and 2.00 ± 0.36 mm, respectively, for posterior implants. A statistically significant difference was observed between the anterior and posterior implants in mean plaque index values. Periodontal outcome parameters at both time points are summarized in Table 1.

**Table 1:** Periodontal outcome parameters at three and six months SD = Standard Deviation. *p < 0.05 statistically significant difference between anterior and posterior implants for plaque index, pain scale (three months), and crestal bone loss (three and six months).

Parameter	Region	3 Months (Mean ± SD)	6 Months (Mean ± SD)
Plaque Index	Anterior	0.426 ± 0.64	0.71 ± 0.46
	Posterior	0.750 ± 0.44	0.37 ± 0.50
Gingival Index	Anterior	0.64 ± 0.49	0.78 ± 0.69
	Posterior	0.37 ± 0.49	0.68 ± 0.47
Probing Depth (mm)	Anterior	1.53 ± 0.45	1.85 ± 0.41
	Posterior	1.65 ± 0.56	2.00 ± 0.36
Crestal Bone Loss (mm)	Anterior	1.46 ± 0.53	1.75 ± 0.37
	Posterior	1.25 ± 0.31	1.65 ± 0.35

Mean pain scale values at three and six months for anterior implants were 0.287 ± 0.46 and 0.14 ± 0.36, respectively; however, pain scores for posterior implants were not statistically significant. A statistically significant difference was observed between anterior and posterior implants in mean pain scale values at three months.

Mean crestal bone loss at three and six months was 1.46 ± 0.53 mm and 1.75 ± 0.37 mm, respectively, for anterior implants and 1.25 ± 0.31 mm and 1.65 ± 0.35 mm, respectively, for posterior implants. A statistically significant difference was observed between anterior and posterior implants in mean crestal bone loss at both time points (p < 0.05).

Mean osseointegration quality (Hounsfield units) immediately after placement, at three months, and at six months was 764.2 ± 130.66, 802.14 ± 124, and 799.2 ± 157.69 HU, respectively, for anterior implants and 655.00 ± 66.53, 706.25 ± 55.36, and 727.00 ± 73.05 HU, respectively, for posterior implants, as detailed in Table 2. As noted in the Discussion, these Hounsfield unit values reflect a relative, device-specific density trend within this study's own CBCT protocol and should not be interpreted against Misch's medical CT-based D1-D4 classification.

**Table 2:** Osseointegration quality - bone density in Hounsfield units (HU) HU = Hounsfield Units (measured on CBCT). Statistically significant difference observed between anterior and posterior implants at all time points (p < 0.05).

Region	Immediate Post-Op (HU)	3 Months (HU)	6 Months (HU)
Anterior Implants	764.2 ± 130.66	802.14 ± 124.00	799.2 ± 157.69
Posterior Implants	655.00 ± 66.53	706.25 ± 55.36	727.00 ± 73.05

A sensitivity analysis excluding the two failed implants is presented in Table 3. Excluding these implants did not materially change reported plaque index, gingival index, probing depth, crestal bone loss, or osseointegration values; however, mean pain scores fell to 0.00 ± 0.00 in both the anterior and posterior groups, since the two failed implants were the only implants in the cohort with any recorded pain.

**Table 3:** Sensitivity analysis excluding the two failed implants Osseointegration (HU), full cohort (as published in Table 2) vs. excluding failures: Anterior immediate 764.2 ± 130.66 vs. 770.00 ± 129.68; 3mo 802.14 ± 124 vs. 800.00 ± 132.10; 6mo 799.2 ± 157.69 vs. 823.08 ± 131.87. Posterior immediate 655.00 ± 66.53 vs. 662.67 ± 74.50; 3mo 706.25 ± 55.36 vs. 714.67 ± 60.81; 6mo 727.00 ± 73.05 vs. 734.00 ± 80.78. *The manuscript text describes posterior pain scores at three and six months as "not statistically significant" without reporting a numeric mean; the case-level dataset indicates a low posterior pain value (approximately 0.06 ± 0.25 at both time points, full cohort), consistent with this description. Excluding the two failed implants leaves periodontal, bone loss, and osseointegration outcomes essentially unchanged; pain falls to zero in both regions, since the two failed implants were the only implants in the cohort with any recorded pain.

Parameter	Region	Full Cohort (n=30)	Excluding Failures (n=28)
Plaque Index 3mo	Anterior	0.426 ± 0.64	0.46 ± 0.66
	Posterior	0.750 ± 0.44	0.80 ± 0.41
Plaque Index 6mo	Anterior	0.71 ± 0.46	0.69 ± 0.48
	Posterior	0.37 ± 0.50	0.33 ± 0.49
Gingival Index 3mo	Anterior	0.64 ± 0.49	0.62 ± 0.51
	Posterior	0.37 ± 0.49	0.40 ± 0.51
Gingival Index 6mo	Anterior	0.78 ± 0.69	0.77 ± 0.73
	Posterior	0.68 ± 0.47	0.67 ± 0.49
Probing Depth 3mo (mm)	Anterior	1.53 ± 0.45	1.62 ± 0.58
	Posterior	1.65 ± 0.56	1.53 ± 0.44
Probing Depth 6mo (mm)	Anterior	1.85 ± 0.41	1.85 ± 0.47
	Posterior	2.00 ± 0.36	1.97 ± 0.30
Pain 3mo	Anterior	0.287 ± 0.46	0.00 ± 0.00
	Posterior	n.s.*	0.00 ± 0.00
Pain 6mo	Anterior	0.14 ± 0.36	0.00 ± 0.00
	Posterior	n.s.*	0.00 ± 0.00
Crestal Bone Loss 3mo (mm)	Anterior	1.46 ± 0.53	1.31 ± 0.38
	Posterior	1.25 ± 0.31	1.33 ± 0.41
Crestal Bone Loss 6mo (mm)	Anterior	1.75 ± 0.37	1.65 ± 0.24
	Posterior	1.65 ± 0.35	1.73 ± 0.37

## Discussion

Dental implants have remarkably transformed the rehabilitation process for edentulous patients. However, successful implant placement in an atrophic maxillary arch critically depends on sufficient alveolar height and width. Overcoming the challenges of maxillary sinus pneumatization and resorbed cancellous bone traditionally requires sinus augmentation, ridge augmentation, or bone grafting-procedures associated with unpredictable results and considerable morbidity. An alternative approach is the use of tilted implants, which maximize cortical bone engagement, increase implant-to-bone contact area, provide primary stability, eliminate prosthetic cantilevers, and improve load distribution. In published studies on tilted implants, implant lengths ranged from 10 to 20 mm [5], with greater length correlating with improved stress distribution [16]. Bone resorption around single tilted implants was found to be higher owing to uneven load distribution; multiple tilted implants are, therefore, preferred for better outcomes. The tilted implant design requires angulated multiunit abutments. Saba stated that angulated abutments during rehabilitation enable buccolingual and mesiodistal alignment to correct imprecise jaw relations [17]. The exact degree of implant inclination was not recorded at the time of surgery in this cohort and could not be retrieved from the CBCT planning records or surgical notes. This omission reflects the original scope of the study, which evaluated implant orientation categorically (axial versus tilted) as a functional alternative to maxillary sinus floor augmentation, following the benchmarking methodology of Aparicio et al. [3]. All tilted implants in the present cohort were placed within the standard clinically accepted range, approximately 15°-45°, to bypass the anterior wall of the maxillary sinus and engage the dense cortical bone of the premaxilla or canine pillar, with the final prosthetic path of insertion corrected using angled multiunit abutments. Barnea et al., cited elsewhere in this Discussion, demonstrated a dose-response relationship between angulation and marginal bone loss, and the absence of per-implant angulation data in the present study is acknowledged as a limitation that prevents direct comparison with that dose-response relationship [5].

Primary stability, defined as the clinical immobility of an implant [18], was achieved in all cases in the present study. Implant success and stability are greatly dependent on bone density and volume, assessed by CBCT - a modality preferred for its low radiation dose and ease of acquisition - and expressed in Hounsfield units. Distinct Hounsfield unit ranges correspond to different tissue types; bone typically falls between 150 and 1,800 HU. Misch et al. classified bone quality as follows: D1 bone >1,250 HU, D2 bone 750-1,250 HU, D3 bone 375-750 HU, and D4 bone <375 HU [19]. These thresholds were established using calibrated medical CT, whereas the Hounsfield unit values in the present study were derived from CBCT, which is device-, protocol-, field-of-view-, and voxel-size-dependent and is not directly interchangeable with medical CT HU. The osseointegration values reported here should therefore be interpreted as a relative, device-specific density trend measured consistently within this study's own CBCT protocol, rather than as a validated classification of bone quality against Misch's medical CT-based thresholds. With this caveat, all implants in the present study demonstrated a stable or improving density trend, measured immediately post-placement and at three and six months. Mean osseointegration values for posterior implants were 655.00 ± 66.53, 706.25 ± 55.36, and 727.00 ± 73.05 HU at the post-operative, three-month, and six-month marks, respectively, and for anterior implants were 764.2 ± 130.66, 802.14 ± 124, and 799.2 ± 157.69 HU at the respective time points. A statistically significant difference was observed between anterior and posterior implants in mean crestal bone loss at three and six months (p < 0.05). Peri-implant radiolucency is associated with clinical mobility and implant failure. In the present study, two implants, one anterior and one posterior, placed in two different patients, were found to be clinically mobile at the six-month follow-up. By the implant success criteria of Albrektsson et al., cited elsewhere in this manuscript, clinical mobility at six months constitutes implant failure rather than a dropout [20]. These two implants were therefore classified as failures, yielding a six-month survival rate of 93.3% (28 of 30 implants).

In the present study, mean crestal bone loss at three and six months was 1.46 ± 0.53 mm and 1.75 ± 0.37 mm, respectively, for anterior implants and 1.25 ± 0.31 mm and 1.65 ± 0.35 mm, respectively, for posterior implants. Agnini et al. reported a mean crestal bone loss of 1.42 ± 0.14 mm in 21 patients over a one-year follow-up [21]. Agliardi et al. found that, over a three-year follow-up, only two of 128 angulated implants failed [22]. Theoretically, tilted implants may exhibit greater marginal bone loss [21] owing to their inclination, which generates five times higher compressive stress at the implant neck and tensile stress on the opposing side. However, this hypothesis was not supported by a meta-analysis conducted by Monje et al. [23], and several prospective studies have reported that longer implants (14-16 mm) demonstrate significantly less bone loss and are preferable in regions of high masticatory load [24]. Zampelis et al. showed that a permanently splinted restoration on distal tilted implants did not increase stress on marginal bone [25]. Calandriello et al. reported lower bone loss with tilted implants, attributed to the position of the implant neck relative to the bone crest - the distal aspect was placed sub-crestally, while the mesial aspect was supra-crestal, facilitating better soft tissue healing [26]. Capelli et al. found that implant tilting did not adversely affect marginal bone loss [24]. In contrast, Almeida et al.'s finite element analysis demonstrated that distal tilting of implants in the all-on-four concept increased stress on the maxillary bone [27]. The mean standard deviation of crestal bone loss in the present study was 0.43 mm at three months and 0.36 mm at six months.

Crespi et al. reported one implant failure out of 48 tilted implants, attributed to peri-implantitis [28]. Cavalli et al. reported a 100% cumulative survival rate for tilted implants in their study [7], applying the implant success criteria recommended by Albrektsson et al. [20]. Success was defined as implant stability; absence of peri-implant radiolucency; and freedom from pain, neuropathies, or infection. Surviving implants are those that remain stable but do not fully meet the success criteria - specifically, marginal bone loss exceeding 1.5 mm in the first year or 0.2 mm annually. Dropout implants are those whose patients were lost to follow-up for any reason, including patient death. Failed implants are those removed for any reason [5]. Barnea et al. reported no significant marginal bone loss around tilted implants; however, bone loss was significantly associated with the degree of angulation, with every 10° increase in tilt contributing to approximately 0.6 mm of additional bone loss [5]. In two patients, two implants were found to be mobile, one in the anterior left maxillary region and one in the right posterior maxillary region. Both were classified as failures rather than as dropouts, consistent with the implant success criteria of Albrektsson et al. [20]. Possible causes of failure include premature or excessive masticatory loading relative to the healing period, inadequate osseointegration, infection, and pain. A literature review by Peñarrocha-Oltra et al. [29] on tilted implant rehabilitation of the atrophic maxilla reported success rates ranging from 92.1% to 100% for 782 implants in 329 patients. Barnea et al. demonstrated in a retrospective study that there was no relationship between implant inclination and peri-implant bone loss over a mean functional loading period of 4.86 ± 3.83 years [5], yielding a 93.1% success rate and supporting distal tilting as an accepted solution for limited bone availability in the posterior region.

The mean pain scale values at three and six months for anterior implants were 0.287 ± 0.46 and 0.14 ± 0.36, respectively. Cavalli et al. found no pain associated with any of the implants in their study [7]. They also reported temporomandibular joint (TMJ)-related pain in two patients and alveolar mucositis in four patients, all of which resolved with treatment. In the present study, pain was assessed using the McGill Pain Questionnaire [14], which evaluates the sensory, affective, and evaluative dimensions of pain experience, and the VAS [15]. Two patients reported pain at the implant site-one in the posterior, and one in the anterior maxilla-which persisted beyond the three-month follow-up but eventually resolved. A statistically significant difference was observed between anterior and posterior implants in mean pain scale values at three months.

Plaque accumulation on tilted implant prostheses necessitates rigorous oral hygiene maintenance. Several authors reported that oral hygiene, plaque, and bleeding scores improved progressively over the first two years of follow-up [30-35]. Lindquist et al. demonstrated that patients with poor oral hygiene had a significantly increased risk of peri-implant bone loss compared with those who maintained high standards of plaque control [36]. Hardt et al. further showed that compromised periodontal health adversely influenced crestal bone loss [37]. Koutouzis et al. found that all periodontally compromised patients in their study maintained high infection control throughout a five-year follow-up, with plaque and mucositis scores as low as 5% [38]. A statistically significant difference was observed between anterior and posterior implants in mean plaque index values, as one patient was unable to maintain adequate oral hygiene. The gingival index scores were non-significant.

Mombelli et al. proposed that probe penetration of 3 mm with a force not exceeding 0.5 N represents a criterion for successful implant osseointegration [12]. Probing depth beyond 5 mm is considered indicative of peri-implantitis. Slutzkey et al. reported that a mean probing depth of 4.20 ± 1.25 mm indicated healthy peri-implant soft tissue [39]. In the present study, probing depth at the final follow-up ranged from 2.0 to 2.5 mm, consistent with the findings of Celletti et al., who reported soft tissue pocket depths of 2.2-2.6 mm [40]. Absolute pocket probing depth alone cannot reliably indicate bone loss or inflammation, as multiple factors, including abutment length and tissue thickness, influence measurements. Increased probing depth does not necessarily correlate with marginal bone loss and may reflect differences in biologic width compared with natural dentition. Krennmair et al. identified complications associated with tilted implants, including mucositis, gingival hyperplasia, and recession, in both anterior and posterior regions [41]. However, none of these complications were statistically significant. Hopp et al. reported biological complications -including infection, fistula, mucositis, peri-implant pathology, and abscess-in association with 182 tilted implants, all of which resolved non-surgically through thorough oral prophylaxis with an ultrasonic scaler [42]. In the present study, mean probing depth values at three and six months were 1.53 ± 0.45 mm and 1.85 ± 0.41 mm, respectively, for anterior implants and 1.65 ± 0.56 mm and 2.00 ± 0.36 mm, respectively, for posterior implants.

The limitations of this study include a relatively small sample size of 30 implants and a short follow-up duration of six months, which may not fully reflect long-term implant behavior and bone remodeling patterns. The absence of a control group with conventionally placed axial implants limits direct comparative conclusions. The sample size of 30 implants was predetermined at the time of institutional ethics committee approval and synopsis submission, based on patient availability during the enrollment window, rather than derived from a formal power calculation.

Several additional methodological limitations are acknowledged. First, the degree of implant inclination was not recorded for individual implants, preventing analysis of the relationship between angulation and outcomes described by other authors. Second, formal normality testing was not performed on the underlying dataset prior to applying parametric and non-parametric statistical tests. Third, implants placed within the same patient were treated as independent observations in the chi-square and paired t-test analyses; because implants in the same patient share bone quality, systemic biology, surgical operator, and loading conditions, this clustering was not accounted for and may affect the precision of the reported p-values. Fourth, implant insertion torque, exact implant dimensions, and the examiner calibration protocol were not systematically recorded and could not be reported. Fifth, osseointegration quality was assessed using CBCT-derived Hounsfield units, which are not directly interchangeable with the calibrated medical CT values underlying Misch's D1-D4 classification; these values should be interpreted as a relative, protocol-specific trend rather than a validated bone quality classification. Finally, two implants that became clinically mobile at six months were initially classified as lost to follow-up rather than as failures; they have been reclassified as failures, yielding a six-month survival rate of 93.3% (28 of 30 implants).

## Conclusions

This study concludes that tilted endosseous implants represent a viable therapeutic option, achieving a six-month survival rate of 93.3% (28 of 30 implants) in this cohort. The technique effectively avoids the need for sinus augmentation and achieves adequate primary stability. Crestal bone loss and periodontal parameters were within an acceptable range over the six-month follow-up period, although anterior crestal bone loss at six months (1.75 mm) approaches the 1.5 mm threshold distinguishing implant survival from success under the Albrektsson criteria, and this should be monitored with longer follow-up. Given the sample size, follow-up duration, and absence of a control group, this study does not support claims of long-term predictability or equivalence to conventional axial implantology.

The present study provides a foundation for similar investigations with larger sample sizes, a greater number of outcome variables, and longer follow-up durations to reach robust conclusions regarding the efficacy, stability, and longevity of tilted implants. Multicenter randomized controlled trials with extended follow-up periods are recommended to further validate the clinical utility of tilted implant rehabilitation in atrophic maxillary arches.
